# Annual progress of clinical research on targeted therapy for nonsmall cell lung cancer in 2022

**DOI:** 10.1002/cai2.56

**Published:** 2023-03-01

**Authors:** Yan Huang, Li Zhang

**Affiliations:** ^1^ State Key Laboratory of Oncology in South China Sun Yat‐sen University Cancer Center Guangzhou P. R. China

**Keywords:** ALK, driver gene‐positive lung cancer, EGFR, rare mutations, targeted therapy

## Abstract

With the rapid development of lung cancer molecular detection and precision therapy, targeted therapy has covered the entire process of diagnosis and treatment of nonsmall cell lung cancer patients. Overall mortality from lung cancer has decreased significantly over the past 20 years, especially since the introduction of targeted drugs in 2013. In 2022, targeted therapy for lung cancer has developed rapidly. The optimization of treatment modes and the exploration of new target drugs such as antibody‐drug conjugates will broaden the selection range of nonsmall cell lung cancer patients with positive driver genes. This article reviews the latest advances in targeted therapy for driver gene‐positive lung cancer in 2022.

## Graphical abstract

The two most frequent alterations in nonsmall cell lung cancer patients were epidermal growth factor receptor and KRAS mutations, accounting for 10%–40% and 25%–32%, respectively. Although the incidence rate is relatively low, other gene mutations including anaplastic lymphoma kinase fusion, MET ex14+, ROS1 fusion, KRAS mutation, and so forth, are also crucial to the survival of patients, and numerous significant pharmacological advances have been produced so far.

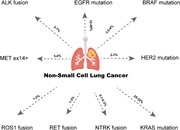

**Abbreviations**
ADC: antibody drug conjugates
ALK: anaplastic lymphoma kinase
BICR: blinded independent central review
CI: confidence interval
CNS: central nervous system
DCR: disease control rate
DFS: disease‐free survival
DOR: duration of response
EGFR: epidermal growth factor receptor
FDA: The USA Food and Drug Administration
HR: hazard ratio
ICI: immune checkpoint inhibitor
MPR: major pathological response
MRD: molecular residual disease
NMPA: The National Medical Products Administration
NSCLC: nonsmall cell lung cancer
ORR: objective response rate
OS: overall survival
PFS: progression‐free survival
PSC: pulmonary sarcomatoid carcinoma
SCLC: small cell lung cancer

## 1 INTRODUCTION

Nonsmall cell lung cancer (NSCLC) accounts for approximately 85% of pulmonary malignancies and is the leading cause of cancer death [1, 2]. Currently, it is estimated that up to 69% of patients with advanced NSCLC harbor druggable mutations in numerous genes [3]. With the rapid development of molecular detection and precision therapy of lung cancer, targeted therapy has significantly improved the survival rate of NSCLC patients with positive driver genes. This article summarizes the progress made in the field of NSCLC‐targeted therapy until the end of 2022 (Table 1).

**Table 1:** Summary of clinical studies in NSCLC with driver gene mutations.

Gene alteration	Study	Therapy	*n*	ORR (%)	DCR (%)	PFS (m)	Reference
EGFR	FURLONG	Furmonertinib	178	89.0	84.0	20.8	[4]
		Gefitinib	179	96.0	93.0	11.1	
	AENEAS	Aumolertinib	214	73.8	72.1	19.3	[5]
		Gefitinib	215	93.0	96.7	9.9	
	NCT02108964	Nazartinib	45	69.0	91.0	18.0	–
	AfaBev‐CS	Afatinib + bevacizumab	49	–	–	16.3	[6]
		Afatinib	50	–	–	16.1	
	WJOG9717L	Osimertinib + bevacizumab	61	–	–	22.1	[7]
		Osimertinib	61	–	–	20.2	
		Osimertinib + PEM + carboplatin	33	90.9	97.0	–	[8]
	CHRYSALIS	Amivantamab + lazertinib	20	100.0	100.0	NE	[9]
EGFR TKI resistance	U31402‐A‐U102	HER3‐DXd	44	39.0	68.0	8.2	[10]
	SAVANNAH	Osimertinib+savolitinib (IHC50 + and/or FISH5+)	193	32.0	61.0	5.3	[11]
		Osimertinib+savolitinib (IHC90 + and/or FISH10+)	108	49.0	74.0	7.1	
	NCT02099058	Osimertinib + Teliso − V	25	58.0	–	–	[12]
		Osimertinib + Teliso − V 1.9 mg/kg	18	67.0	–	–	
	ORIENT‐31	Sintilimab + IBI305 + platinum‐based chemotherapy	158	48.1	86.1	7.2	[13]
		Sintilimab + platinum‐based chemotherapy	158	34.8	81.6	5.5	
		Platinum‐based chemotherapy	160	29.4	75.6	4.3	
EGFR Ex20ins	WU‐KONG1 WU‐KONG2 WU‐KONG6	Sunvozertinib	84	52.4	–	–	[14]
	POSITION20	High dose osimertinib	25	28.0	–	6.8	[15]
EGFR G719X, S768I and L861Q mutations	ARTICUNO	Osimertinib (G719X)	19	50.0	89.0	11.0	[16]
		Osimertinib (L861X)	15	50.0	86.0	9.0	
		Osimertinib (S768I)	11	55.0	91.0	17.0	
ALK	CROWN Asian	Lorlatinib	59	78.0	–	NR	[17]
		Crizotinib	61	57.4	–	11.1	
	J‐ALEX	Alectinib	103	–	–	34.1	[18]
		Crizotinib	104	–	–	10.2	
	NCT03909971	lorlatinib (cohort 1: previous crizotinib)	67	70.1	82.0	NR	[19]
		lorlatinib (cohort 2: one ALK TKI other than crizotinib [±prior crizotinib])	42	47.6	61.9	5.6	
MET14 exon skipping mutation	NCT02897479	Savolitinib	70	47.1	81.4	6.9	[20]
	VISION	Tepotinib (cohort C)	161	54.7	80.1	13.8	[21]
	GLORY	SCC244	73	60.9	82.6	7.6	[22]
HER2 mutation	DESTINY‐Lung02	T‐DXd 5.4 mg/kg Q3W	52	53.8	90.4	–	[23]
		T‐DXd 6.4 mg/kg Q3W	28	42.9	92.9	–	
BRAF V600 mutation	NCT04452877	Dabrafenib + trametinib	18	75	–	NR	[24]
RET fusion	ARROW	Pralsetinib (prior platinum treatment)	130	82	–	16.4	[25]
		Pralsetinib (no prior systemic treatment)	107	83	–	12.6	
	LIBRETTO‐001	Selpercatinib (treatment‐naïve)	69	84.1	–	22.0	[26]
		Selpercatinib (previous platinum chemotherapy)	247	61.1	–	24.9	
	LIBRETTO‐321	Selpercatinib (treatment‐naïve)	8	87.5	–	NR	[27]
		Selpercatinib (pretreated)	18	61.1	–	NR	
KRAS	CodeBreaK 200	Sotorasib	169	28.1	82.5	5.6	[28]
		Docetaxel	151	13.2	60.3	4.5	
	KRYSTAL‐1	MRTX849	112	43	80	6.5	[29]
ROS1 fusion	EUCROSS	Crizotinib	34	70	90	19.4	[30]
	STARTRK‐1, STARTRK2, ALKA‐372‐001 (EudraCT 2012‐000148‐8)	Entrectinib	172	67.4	86.7	16.8	[31]
		Entrectinib (first‐line cohort)	67	68.7	79.1	17.7	
	NCT03972189	Unecritinib	111	78.4	87.4	15.6	[32]
NTRK gene	NCT02576431 NCT02122913	Larotrectinib	26	83	100	NR	[33]
Fusion in a pan‐cancer setting	STARTRK‐1, STARTRK2, ALKA‐372‐001 (EudraCT 2012‐000148‐8)	EntrectinibA	31	64.5	–	–	[34]
	STARTRK 2	Entrectinib (mainland China, Hong Kong, Taiwan)	21	81	85.8	30.3	[35]

Abbreviations: ALK, anaplastic lymphoma kinase; DSR, disease control rate; EGFR, epidermal growth factor receptor; NSCLC, nonsmall cell lung cancer; ORR, objective response rate; PFS, progression‐free survival; TKI, tyrosine kinase inhibitor.

## 2 EPIDERMAL GROWTH FACTOR RECEPTOR‐MUTATED NSCLC

Epidermal growth factor receptor (EGFR) is a protein that drives cell survival, proliferation, and spread. Certain mutations in EGFR lead to protein activation, and some of these mutations are known to be susceptible to agents that bind to and inhibit the protein. The most common EGFR mutations in NSCLC often referred to as “EGFR‐sensitizing mutations,” are deletions of exon 19 (19Del) and a single amino acid substitution L858R in exon 21 (L858R), which together account for up to 85% of observed EGFR mutations in NSCLC. Rare mutations account for the remaining 15% of EGFR mutations in NSCLC and include point mutations, deletions, and insertions within exons 18–25 of the EGFR gene, such as Ex20ins, G719X, S768I, and L861Q mutations [36]. EGFR tyrosine kinase inhibitors (TKIs) have been used as adjuvant therapy after tumor resection as multiple randomized trials have demonstrated their effectiveness, as first‐line treatment for EGFR exon 19 deletions or exon 21 L858R mutations of NSCLC patients. Therefore, research progress on EGFR mutations is mainly focused on perioperative targeted therapy, first‐line therapy, and management of acquired resistance to EGFR TKIs. As for rare EGFR mutations, more novel drugs are showing promising results.

### 2.1 Perioperative targeted therapy

In the neoadjuvant setting, NEOS is a prospective, multicenter, single‐arm study to evaluate the efficacy and safety of osimertinib as neoadjuvant therapy for resectable epidermal growth factor receptor‐mutated (EGFRm) (19Del/L858R) lung adenocarcinoma. Forty patients received osimertinib 80 mg orally daily for 6 weeks before undergoing surgery. The objective response rate (ORR) was 71%, the disease control rate (DCR) was 100%, and the major pathological response rate (MPR) was 11% [37]. This is the largest sample to evaluate osimertinib as neoadjuvant therapy. Another study evaluating osimertinib with or without chemotherapy versus chemotherapy alone as neoadjuvant therapy in patients with resectable EGFR‐mutated NSCLC NeoADAURA (NCT04351555) is ongoing. It is hoped that the addition of chemotherapy will lead to better MPR rates.

In terms of adjuvant therapy, updated results from the ADAURA trial were represented at the 2022 ESMO Conference. Median follow‐up was 44.2 months for osimertinib and 19.6 months for placebo. The median disease‐free survival (DFS) for Stage II–IIIA patients was 65.8 months (95% confidence interval [CI]: 54.4‐NC) in the osimertinib arm and 21.9 months (95% CI: 16.6–27.5) in the placebo arm (hazard ratio [HR] for disease recurrence or death = 0.23). The 4‐year DFS rate for Stage II–IIIA patients in the osimertinib arm was 70%; the median DFS rate for the overall population in the osimertinib arm was 65.8 months compared with 28.1 months in the placebo arm [38]. Results of this update demonstrate a sustained, clinically meaningful improvement in DFS compared with placebo in the adjuvant setting after complete tumor resection with curative intent in patients with early‐stage (IB, II, and IIIA) EGFRm NSCLC. Meanwhile, the EVAN study was published, demonstrating for the first time a clinically meaningful overall survival (OS) improvement with adjuvant erlotinib compared with chemotherapy in Stage III EGFRm NSCLC, with median OS and 5‐year OS rates of 84.2 months and 84.8%, respectively [39]. The above‐updated results further confirm the role of adjuvant targeted therapy. When we see that Stage III patients can achieve such excellent results, we should think that Stage I patients can achieve a higher 5‐year survival rate than patients with Stage III. This is may be why more and more pharmaceutical companies and specialists are exploring treatments for patients with Stage I lung cancer, such as ADAURA2 (NCT05120349).

In the precision‐assisted setting, a study conducted by Wu et al. [40] demonstrated that molecular residual disease (MRD) detection can predict the prognosis of NSCLC patients after surgery. Two hundred and sixty‐one patients with Stage I–III NSCLC were enrolled and 913 peripheral blood samples were successfully tested by MRD assay. In the population, only six patients (3.2%) with undetectable longitudinal MRD relapsed, resulting in a negative predictive value of 96.8%. Correspondingly, the positive predictive value of longitudinally detectable MRD was 89.1%. This study demonstrates the prognostic value of longitudinally undetectable MRD in NSCLC patients after radical surgery for defining a potentially curable population in localized NSCLC. This is a promising direction for precision therapy for early‐stage lung cancer patients.

### 2.2 First‐line therapy

#### 2.2.1 EGFR TKI monotherapy

In the Chinese Phase III trial FURLONG [4], compared with gefitinib, the third‐generation EGFR‐TKI furmonertinib in the first‐line treatment of NSCLC with sensitive EGFR mutations can significantly prolong the median progression‐free survival (PFS) (19.3 months vs. 9.9 months, HR = 0.46) and central nervous system PFS (20.8 months vs. 9.8 months, HR = 0.40). Similarly, other third‐generation EGFR‐TKIs, aumolertinib, and nazartinib showed promising results in the AENEAS study [5] and NCT02108964, respectively, with the median PFS of aumolertinib and nazartinib being 19.3 and 18.0 months, respectively. These studies have once again verified the value of third‐generation EGFR‐TKI recommended for the first‐line treatment of NSCLC with sensitive EGFR mutations. Nearly 20 months has become the limit of third‐generation EGFR TKIs, so a series of combination therapies have followed.

#### 2.2.2 EGFR‐TKIs combination therapy

As for how to optimize the first‐line treatment, the combination of EGFR TKIs is mainly explored. The impact of EGFR TKIs plus antiangiogenic therapy, chemotherapy, or bispecific monoclonal antibodies remains in the spotlight.

For EGFR TKI combined with antiangiogenic therapy, AfaBev‐CS study was a randomized, open‐label, multicenter Phase II trial comparing afatinib plus bevacizumab versus afatinib alone as first‐line treatment for EGFR‐mutated advanced NSCLC [6]. This study failed to show an improvement in PFS with afatinib plus bevacizumab versus afatinib monotherapy (16.3 vs. 16.1 months, HR = 0.87, *p* = 0.55). Similarly, a Phase II WJOG9717L study [7] showed that first‐line treatment with osimertinib plus bevacizumab failed to improve median PFS in patients with sensitive EGFR mutations (22.1 vs. 20.2 months, HR = 0.86, *p* = 0.21).

For EGFR TKIs plus chemotherapy, the Phase II OPAL study [8], the ORR of osimertinib combined with platinum and pemetrexed was 90.9%, and the DCR was 97%, which will be further evaluated in Phase III study FLAURA2 (NCT04035486).

For EGFR TKIs plus bispecific monoclonal antibodies, at the 2022 WCLC conference, amivantamab plus lazertinib showed 100% ORR in untreated EGFR‐mutated NSCLC. The median follow‐up was 22.3 months, median PFS was not reached, and the safety was consistent with other reports [9].

#### 2.2.3 Management of acquired resistance to EGFR TKIs

Despite the efficacy of highly potent EGFR TKIs, acquired resistance is inevitable and remains a critical unsolved challenge. Broadly speaking, acquired resistance can be classified as “on‐target” or “off‐target.” For “on‐target,” the most common T790M mutation is the dominant mechanism of acquired resistance to first‐/second‐generation EGFR‐TKIs, and C797S mutation is associated with the common third‐generation EGFR‐TKI resistance. For “off‐targets,” such as MET amplification/overexpression, HER2 amplification, BRAF mutation, RET fusion, small cell lung cancer (SCLC) transformation, and so forth, there have been a lot of research and exploration in this field. In the following sections, we will highlight recent advances in drug resistance research.

##### 2.2.3.1 On‐target resistance

###### 2.2.3.1.1 EGFR C797S mutation

Several fourth‐generation EGFR‐TKIs, such as BLU‐701, BLU‐945, JIN‐A02, LS‐106, and BBT‐176, have been developed to overcome EGFR C797S resistance. The robust in vivo and in vitro antitumor activity in osimertinib‐resistant NSCLC models is promising and warrants further investigation. Whether the fourth‐generation EGFR TKI can really change clinical practice may need to be further verified through large‐scale clinical studies, and we also need to consider the degree of benefit and adverse events. At the 2022 ASCO meeting, there is a Phase I/II, open‐label SYMPHONY study (NCT04862780) to evaluate the safety, pharmacokinetics, and anticancer properties of BLU‐945 monotherapy or in combination with osimertinib in patients with C797S mutation [41].

###### 2.2.3.1.2 Antibody‐drug conjugates (ADCs)

The first human study of TROP2 ADC datopotamab deruxtecan (Dato‐DXd) showed an ORR of 35% and a median duration response of 9.5 months in the treatment of NSCLC with actionable genomic alterations [42]. According to the Phase I U31402‐A‐U102 study, HER3‐DXd achieved an ORR of 39%, a DCR of 68%, and a median PFS of 8.2 months in patients with advanced EGFR‐mutated NSCLC who had previously received osimertinib and platinum‐based chemotherapy. The most common Grade ≥ 3 treatment‐related adverse events (TRAEs) was hematologic toxicity [10]. The Phase III study HERTHENA‐Lung02 (NCT05338970) is currently ongoing.

##### 2.2.3.2 Off‐target resistance

###### 2.2.3.2.1 MET amplification/overexpression resistance

SAVANNAH is a Phase II single‐arm trial of osimertinib plus savolitinib in osimertinib‐resistant patients with MET overexpression and/or amplification [11]. The ORR was 32% (62/193) in the overall population (IHC 50% and/or FISH 5 + status) and 49% (52/108) in patients with IHC90 + and/or FISH10 + status (i.e., higher levels of proteins and/or mRNAs), and the median PFS was 5.3 months and 7.1 months, respectively. Improved outcomes were observed in patients with higher protein and/or mRNA levels, confirming the need for appropriate MET biomarker‐based patient selection in this population. The efficacy of savolitinib plus osimertinib is being further assessed in the Phase III SAFFRON (NCT05261399) study. Another novel drug is telisotuzumab vedotin (Teliso‐V), a humanized monoclonal antibody targeting c‐Met. In a Phase I/Ib study (NCT02099058) [12], Teliso‐V plus osimertinib was effective in treating advanced NSCLC with MET overexpression after osimertinib resistance. Twenty‐five patients received Teliso‐V (1.6 mg/kg, *n* = 7; 1.9 mg/kg, *n* = 18) plus osimertinib. The ORR was 58% in all patients and 67% at 1.9 mg/kg in NSCLC patients with c‐Met overexpression. Teliso‐V plus osimertinib was well tolerated, with no dose‐limiting toxicities reported. The major obstacle of MET in the future is detection, and detection methods and cut‐off values need to be clarified.

###### 2.2.3.2.2 Combination therapy with immune checkpoint inhibitors (ICIs)

The second interim analysis of the Phase III ORIENT‐31 study [13] reported at ESMO 2022 revealed that platinum‐based chemotherapy plus sintilimab with or without a VEGF inhibitor significantly improved PFS compared with chemotherapy monotherapy. The median PFS was 7.2, 5.5, and 4.3 months for the four‐drug combination group (Arm A), sintilimab plus chemotherapy (Arm B), and chemotherapy (Arm C), respectively. The safety profile of the treatment was acceptable, and no new safety concerns were identified.

#### 2.2.4 Rare mutation

##### 2.2.4.1 EGFR Ex20ins

EGFR exon 20 insertion mutations (EGFR 20ins), account for 4%–12% of EGFR‐mutated NSCLC [43]. Amivantamab, a bispecific antibody targeting EGFR and MET, is currently FDA‐approved for the treatment of NSCLC with EGFR Ex20ins mutations after progression on chemotherapy. A randomized Phase III PAPILLON study (NCT04538664) compared amivantamab plus carboplatin‐pemetrexed versus carboplatin‐pemetrexed in patients with EGFR exon 20ins mutant NSCLC.

Sunvozertinib (DZD9008) is an oral EGFR exon 20ins inhibitor developed by a Chinese company. The pooled data of the global multicenter trials (WU‐KONG1, WU‐KONG2, and WU‐KONG6) [14] reported at the 2022 WCLC showed that the ORR of sunvozertinib in 84 chemotherapy failure patients with EGFR exon 20ins mutation advanced NSCLC was 52.4% and 44% for patients with brain metastases (*n* = 25). The most common Grade ≥ 3 treatment emergent adverse events were elevated creatine kinase (9.2%) and diarrhea (5.5%). The US FDA approved sunvozertinib as a breakthrough treatment in January 2022. The POSITION20 trial which explored high‐dose osimertinib in patients with EGFR exon 20 mutation‐positive advanced NSCLC, showed modest antitumor activity with a confirmed ORR of 28%, PFS of 6.8 months, and acceptable toxicity [15].

##### 2.2.4.2 EGFR G719X, S768I, and L861Q mutations

Ten to twenty percent of NSCLC patients carry uncommon EGFR mutations that respond differently to various EGFR TKIs [44]. Edward et al. reported that the ORRs of afatinib in NSCLC patients with G719X, S768I, and L861Q mutations were 63.4%, 62.5%, and 59.6%, respectively [45]. According to an Italian retrospective study (ARTICUNO trial) [16] presented at the 2022 ESMO Congress, the ORRs of osimertinib in NSCLC with G719X, L861X, and S768I mutations were 50%, 50%, and 55%, respectively, and PFS 11, 9, and 17 months, respectively. The NCCN guidelines currently prioritize afatinib and osimertinib for the treatment of NSCLC with EGFR S768I, L861Q, and/or G719X mutations [46].

## 3 ANAPLASTIC LYMPHOMA KINASE FUSION

In China, 3%–7% of NSCLC patients have an anaplastic lymphoma kinase (ALK) fusion, which is a common driver of the disease [47].

In the ALEX trial [48], the 5‐year survival rate of first‐line aletinib was 62.5%, while the 5‐year OS rate of crizotinib was 45.5% (HR = 0.67, *p* = 0.0376), indicating that the second‐generation ALK‐TKI aletinib prolongs survival.

At the 2022 ESMO Conference, data from the Asian subgroup of the Phase III CROWN study [17] demonstrated that the median PFS (as assessed by BICR) was not reached in the loratinib group and 11.1 months in the crizotinib group (HR = 0.40; 95% CI: 0.23–0.71), and the 3‐year PFS rates were 61% and 25%, respectively, further confirming the efficacy of the third‐generation ALK‐TKI. However, in the J‐ALEX study, compared with crizotinib, alectinib did not prolong the OS of Japanese patients (HR 1.03, 95% CI: 0.67–1.58; *p* = 0.9105), which is likely to be confounded by cross‐treatment. The 5‐year OS rate was 60.9% (95% CI: 51.4–70.3) for alectinib and 64.1% (95% CI: 54.9–73.4) for crizotinib [18]. Alectinib resulted in long‐term survival in patients with advanced ALK‐positive NSCLC, and although no OS extension was observed relative to crizotinib, it remains the standard of care for patients with advanced ALK‐positive NSCLC.

In March 2022, a Phase II study (NCT03909971) reported results of lorlatinib in Chinese patients with previously treated ALK‐positive advanced NSCLC. A total of 47 patients were previously treated with crizotinib (70.1%, 95% CI: 57.7–80.7, *p* < 0.0001) and 20 patients were previously treated with an ALK‐TKI other than crizotinib (47.6%, 95% CI: 32.0–63.6) achieved an objective response [19]. The National Medical Products Administration (NMPA) approved loratinib in April 2022 for the treatment of patients with ALK mutation‐positive advanced NSCLC who were previously treated with an ALK inhibitor.

At present, ALK‐TKI therapy presents a positive pattern of “three generations under one roof.” More efficient and less toxic new‐generation ALK inhibitors and combination therapy are expected to further improve the survival of ALK‐positive patients.

## 4 MET14 EXON SKIPPING MUTATION (METEX14+)

MET14 exon skipping mutations (METex14+) are considered to be independent oncogenes of lung cancer, with an overall incidence of 3%–4% in NSCLC [49]. Currently, several MET TKIs (capmatinib and tepotinib) are approved globally for the treatment of METex14+ NSCLC. The only MET inhibitor approved in China is savolitinib.

ELCC 2022 updated final OS and subgroup analysis of savolitinib in METex14+ NSCLC patients. In 36% of patients with pulmonary sarcomatoid carcinoma, 21% of patients with brain metastases and 60% of patients who had previously received savolitinib, ORR was 47.1%, PFS was 6.9 months and median OS was 12.5 months; The median PFS was 7.0 months and the median OS was 17.3 months in patients with non‐PSC NSCLC; the median PFS was 7.0 months and the median OS was 17.7 months in patients with brain metastases [20].

The Phase II VISION trial (NCT02864992) included patients who had previously received less than second‐line therapy and allowed recruitment of patients with asymptomatic brain metastases. Tepotinib‐treated METex14+ patients had an ORR of 54.7%, a median PFS of 13.8 months and a median OS of 18.8 months [21]. A new drug application was submitted for tepotinib in China in March 2022.

In the GLORY study [22], presented at the 2022 AACR Congress, patients who had previously received no more than two systemic therapies, such as chemotherapy and immunotherapy, received glumetinib (SCC‐244). The ORR was 60.9% for overall patients, 66.7% for treatment‐naïve patients, and 51.9% for previously treated patients. A priority review for SCC‐244 was conducted in China on February 25, 2022.

## 5 HER2 MUTATION

In January 2022, the DESTINY‐Lung01trial demonstrated clinically significant antitumor activity of trastuzumab deruxtecan (also known as T‐DXd or Enhertu) in patients with HER‐2‐mutant NSCLC. A total of 91 patients were enrolled, the ORR was 55%, median duration of response (DoR) was 9.3 months, median PFS was 8.2 months, and the median OS was 17.8 months [23]. Also, in the Phase II clinical trial DESTINY‐Lung02 [50], T‐DXd in metastatic NSCLC with HER2 mutations showed an ORR of 53.8% and a median DoR of 8.7 months. On August 11, 2022, the FDA approved T‐DXd for the treatment of advanced NSCLC with HER2 mutations.

## 6 BRAF V600 MUTATION

The incidence of BRAF mutations in NSCLC is 3.5%–4%, and BRAF V600E accounts for about 50% of all BRAF mutations [51].

At the 2022 WCLC, results of the first Phase I trial conducted in Chinese lung cancer patients with BRAF mutations were presented. A 75% ORR and a manageable safety profile were demonstrated with dabrafenib plus trametinib [24].

The NMPA‐approved dabrafenib combined with trametinib for the treatment of BRAF V600 mutation‐positive NSCLC in March 2022, becoming the first approved combination therapy in China.

## 7 RET FUSION NSCLC

The frequency of RET fusions in NSCLC is 1%–3% [52]. According to the ARROW study reported at ESMO 2022, pralsetinib had an ORR of was 83% in treatment‐naïve patients and 82% in patients who had previously received platinum‐based chemotherapy, while median PFS was 12.6 and 16.4 months, respectively. These data suggest that pralsetinib is able to provide clinical benefit in the majority of NSCLC patients with RET fusions [25].

Based on the updated data from the LIBRETTO‐001 trial, selpercatinib had an ORR of 84.1% in 69 treatment‐naïve patients, with a median PFS of 22.0 months and a median DoR of 20.2 months; in 247 patients who had previously received platinum‐based therapy, the ORR was 61.1%, while the median PFS was 24.9 months and the median DoR was 28.6 months [26]. The LIBRETTO‐321 study reported ORRs of 87.5% and 61.1% for selpercatinib in untreated and previously treated patients, respectively, confirming the satisfactory efficacy and safety of selpercatinib in Chinese patients with RET fusion NSCLC [27].

Currently, pralsetinib and selpercatinib are both approved in the United States and abroad for the treatment of patients with RET fusion NSCLC.

## 8 KRAS MUTANT NSCLC

KRAS mutations are the second most common mutation type in NSCLC, after EGFR mutations [53]. KRAS mutations were most commonly found in G12C (39%), G12V (21%), and G12D (17%).

Sotorasib (AMG510) is the first FDA‐approved KRAS^G12C^ inhibitor and has also been identified as a “breakthrough therapy drug” by the NMPA.

As reported in the Phase III CodeBreak 200 study, sotorasib showed better ORR (28.1% vs, 13.2%) and longer median PFS (5.6 vs. 4.5 months, HR = 0.66, *p* = 0.002) than docetaxel in KRAS^G12C^‐mutated NSCLC patients, but there was no significant difference in OS [28].

At the 2022 ASCO conference, Spira et al. reported the results of Phase I/II KRYSTAL‐1 (NCT03785249) trial [29]. Adagrasib (MRTX849) was indicated for the treatment of KRAS^G12C^ mutated NSCLC patients who had received platinum‐based chemotherapy and anti‐PD‐1/PD‐L1 therapy. The ORR was 42.9%, DCR was 79.5%, DoR was 8.5 months, median PFS was 6.5 months, and median OS was 12.6 months.

Many drugs targeting KRAS mutations are still in the early stages of development. As reported at the WCLC conference in 2022, a Phase I study of GDC‐6036 and D‐1553 monotherapy demonstrated antitumor activity in patients with KRAS^G12C^‐mutated NSCLC [54].

## 9 ROS1 FUSION

Rearrangements of the ROS1 gene are found in 1%–2% of NSCLC. The 2022 ASCO annual meeting reported a prospective EUCROSS trial, which revealed that crizotinib was very effective in ROS1 fusion NSCLC, with an ORR of 70%, a median PFS of 19.4 months and a 4‐year OS rate of 55.0% [30].

Entrectinib has been approved internationally and domestically for the treatment of patients with ROS1 fusion‐positive NSCLC. According to the WCLC 2022 report, the ORR of entrectinib in patients with ROS1 fusion NSCLC was 68.7%, the median DoR was 35.6 months, the median PFS was 17.7 months, and the median OS was 47.7 months. Entrectinib has shown durable efficacy [31].

Unecritinib (TQ‐B3101), as the first‐line therapy for ROS1 fusion‐positive Chinese NSCLC patients, showed an ORR of 78.4% and a median PFS of 15.6 months, suggesting good efficacy and manageable toxicity [32].

## 10 NTRK GENE FUSIONS IN THE PAN‐CANCER CONTEXT

The prevalence of NTRK fusions in NSCLC patients is estimated to be between 0.1% and 0.3% [55]. Both larotinib and entrotinib have been approved by the FDA and NMPA for the treatment of NTRK gene‐positive solid tumors.

At WCLC 2022, updated data on long‐term follow‐up of patients with NTRK gene fusions treated with larotinib were presented. The ORR was 83% and the median OS was as high as 40.7 months. For patients with brain metastases, the ORR was 80%, median PFS was 9.9 months, and median OS was 19.4 months [33].

A meta‐analysis [34] showed an ORR of 61.3% in NTRK fusion‐positive patients across 17 different solid tumor types treated with entrectinib. The median DoR, PFS, and OS were 20.0 months (95% CI: 13.2–31.1), 13.8 months (95% CI: 10.1–20.0), and 37.1 months (95% CI: 27.2–not estimable), respectively. Among them, 31 patients with NSCLC had a similar efficacy, with an ORR of 64.5%.

Data from Chinese subgroup of the STARTRK‐2 trial released at the ELCC Congress in 2022 showed that the ORR of 21 patients with NTRK fusion‐positive solid tumors treated with larotinib was 81.0%, and the median PFS was 30.3 months [35].

## 11 FUTURE DIRECTION

Although there are many studies on classic mutations such as EGFR and ALK, further optimization of treatment strategies and accurate screening of patients will be the focus in the future.

With the widespread development of high‐throughput sequencing technology, new therapies for refractory mutation subtypes, such as EGFR 20ins and KRAS mutations, can be developed by exploring uncommon targets and developing new targeted drugs.

As a powerful and precise antitumor drug, ADCs have emerged as the most promising therapeutic approach. In the future, further exploration of combination therapy and screening of effective biomarkers will help the development of ADCs for precision therapy.

Combination therapy with ICIs holds promise for patients with rare genetic mutations or resistance to TKI therapy, and more evidence is expected to emerge in the future.

## 12 CONCLUSION

With the development of precision medicine, lung cancer treatment has entered the era of individualized precision. Further efforts in patient identification, treatment optimization, and in‐depth studies of resistance to third‐generation TKIs are required to maximize patient benefit.

## AUTHOR CONTRIBUTIONS

**Yan Huang**: data curation (supporting); formal analysis (supporting); Investigation (supporting); methodology (supporting); writing—original draft (supporting). **Li Zhang**: conceptualization (lead); data curation (lead); formal analysis (lead); investigation (lead); methodology (lead); resources (lead); supervision (lead); writing—original draft (lead); writing—review and editing (lead).

## CONFLICT OF INTEREST STATEMENT

The authors declare no conflict of interest.

## ETHICS STATEMENT

Not applicable.

## INFORMED CONSENT

Not applicable.

## Data Availability

The data that support the findings of this study are openly available.
